# Military Personals

**Published:** 1918-11

**Authors:** 


### MILITARY PERSONALS

To Camp Custer, Capt. J. J. Vorbett, Syracuse.

To Camp Greene, Lieut. E. M. Trench, Buffalo.

To Camp A. A. Humphreys, Lieut. A. C. Abbott, Syracuse.

To Camp Dix, Capt. H. G. Germer, Canastota.

To Camp Jackson, Capt. Harry Weed, Buffalo.

To Fort Benjamin Harrison, Lieut. W. L. McCanty, Buffalo.

To Fort Oglethorpe, Lieuts. F. P. Schenkelberger, Gowanda; G. C. Barone, Buffalo; D. N. Cott.

To Fort Snelling, Lieut. I. A. Wentworth, Clifton Springs.

To Hoboken, Lieuts. W. H. Jones, J. P. LaDuca, Buffalo.

To New Haven, Lieut. J. L. Mangano, Buffalo.

To Rockefeller Institute, Lieut. D. Jung, Buffalo.

To Baltimore, Capt. D. S. Childs, Syracuse.

To Camp Beauregard, Capt. G. Priestman, Willard.

To Camp Shelby, Lieut. H. A. Patterson, Buffalo.

To Camp Wheeler, Capt. E. W. Ayers, Alfred.

To Fort Niagara, Capt. N. B. Ford, Owasco.

To Mineola, Capt. E. L. Hazeltine, Jamestown.

To Camp Crane, Capt. W. R. Thomson, Warsaw; Lieut. G. G Davis, Arcade.

To Middletown, Pa., Lieut. M. H. Goldberg, Buffalo.

Honorably discharged on account of physical disability, Lieut. W. W. Millias, Rome.

Dr. T. W. Price of Medina was wounded in action on the Vosges front in France on Aug. 24.

Dr. Arthur E. Mitten, formerly of Lockport, has been taken prisoner by the Germans and is in a prison camp near Karlsruhe. Dr. Mitten with other medical corps officers attached to Wisconsin regiments were forward to establish first aid stations. They were in the midst of the battle area and a shell struck near their machine, blowing it to pieces. It was not known whether Mitten was killed. Doubt as to his safety has been cleared up by the word from the Red Cross. Dr.

Mitten is a graduate of the University of Buffalo. After graduation he took up practice in Milwaukee. His wife lives there.

Dr. William Ward Plummer, of Buffalo, now in France, had been promoted to a majority on August 28th. He is now consultant to twelve base hospitals in the Saint Mihiel region. Dr. Plummer went abroad last October as a captain in a special unit of orthopedic surgeons headed by Dr. Goldwaite of Boston. He had attained distinction in that branch of surgery. For eight months Dr. Plummer was head surgeon in a big hospital in Leeds, England, and in the early summer was transferred to Southern France, where he had charge of a special training camp. In August he was called to the Saint Mihiel region and his promotion followed.

Major General Ireland has had his nomination as Surgeon General approved by the Senate.

Major Francis Fronczak of Buffalo represented the Red Cross when General Polo was sworn in as commander in chief of the Polish Army. The ceremony took place in the presence of the first Polish division, 80 per cent of whose members are Poles from U. S. General Polo and a number of his men deserted the Austrians last winter and escaped from Russia by way of Murmansk. This division is complete in every particular and has cavalry, artillery and hospital detachments. All the equipment came from U. S.

